# Do government subsidies lead to TFP losses? A perspective on the formation of zombie firms

**DOI:** 10.1371/journal.pone.0338612

**Published:** 2025-12-18

**Authors:** Ruying Chen, Danyu Liu, Lanyu Wu, Donghua Yu

**Affiliations:** 1 School of Business, Linyi University, Linyi, Shandong, China; 2 School of Digital Economy and Management, Guangxi University of Foreign Languages, Nanning, Guangxi, China; 3 School of Economics, Shandong University, Ji’nan, Shandong, China; Southern Illinois University Carbondale, UNITED STATES OF AMERICA

## Abstract

This study examines how government subsidies affect aggregate total factor productivity (TFP) through the formation of zombie firms in China. Using panel data of A-share listed firms from 2007 to 2022 and industry–province–year aggregates, we analyze both firm-level dynamics and aggregate productivity outcomes. The results show that subsidies reduce the exit probability of low-productivity firms and significantly increase the likelihood of zombie firm formation. Although the prevalence of zombie firms fluctuates over time, it remains a persistent challenge. At the aggregate level, subsidies exert a negative impact on TFP, with zombie firm prevalence serving as a key mediating channel. The mediating effect is particularly strong in industries with a higher share of state-owned enterprises (SOEs), whereas regional differences stem from distinct mechanisms: subsidies more strongly induce zombie formation in Central–Western China, while the productivity drag of zombies is larger in the more competitive Eastern region. These findings highlight the unintended productivity costs of subsidy policies and underscore the need for more targeted and efficiency-oriented intervention.

## Introduction

Governments frequently employ subsidies as policy instruments to stimulate economic growth across various sectors [1,2]. This approach is particularly crucial in developing countries, where firms typically experience pronounced market failures and externalities relative to those in developed economies, necessitating substantial government intervention through subsidy policies [3,4]. In many cases, these subsidies are deliberately directed toward high-risk but socially or strategically important sectors such as new energy vehicles, semiconductors, or advanced batteries, where breakthrough innovations require a long time to materialize. In the context of rapid economic expansion, the Chinese government has implemented targeted subsidy policies aimed at enhancing enterprises’ competitiveness and innovative capabilities, thereby promoting the modernization and structural upgrading of multiple industrial sectors. Consequently, the impact of government subsidies has attracted extensive scholarly attention. Numerous studies have examined this topic from diverse perspectives, such as alleviating market failures, fostering innovation, and optimizing resource allocation [2,5,6]. However, a substantial body of literature also highlights deficiencies in governmental processes for selecting subsidy recipients, underscoring potential adverse consequences of subsidy allocation. A prominent negative outcome associated with poorly designed subsidy mechanisms is the proliferation of zombie firms [7]. Hence, investigating how government subsidies affect TFP, particularly in relation to corporate exit behaviors, holds significant theoretical and practical value.

This study examines the impact of Chinese government subsidies on aggregate TFP through the formation of zombie firms. Specifically, it analyzes how subsidies influence firms’ exit behaviors and how they contribute to the formation and proliferation of zombie firms. Additionally, this research evaluates the current landscape of zombie firms in China, examines heterogeneous subsidy effects across ownership types and regions, and investigates the mediating role of zombie firms in the subsidy–TFP relationship.

The remainder of this paper is structured as follows. The Literature Review section reviews the relevant literature. The Theoretical Analysis section presents the mechanisms through which government subsidies affect firm exit, zombie firm formation, and their subsequent impact on aggregate TFP. The Empirical Models Setting and Data Description section introduces the empirical model and describes the data. The Empirical Results and Discussions section discusses the empirical results and their implications. Finally, the Concluding Remarks and Policy Implications section summarizes the key findings and offers policy recommendations.

## Literature review

### Impact of government subsidies on TFP

Government subsidies represent a commonly used policy tool globally, intended to correct market failures, guide resource allocation, and foster technological innovation [8–10]. Typical subsidy mechanisms include direct financial assistance, tax incentives, research and development (R&D) grants, and interest rate subsidies on loans [11]. Existing studies present diverse views regarding the overall effectiveness of subsidy policies. In particular, scholars have extensively debated how government subsidies influence TFP.

Several studies indicate that subsidies significantly enhance TFP [12]. The literature identifies several mechanisms underlying these positive effects. First, subsidies reduce capital costs and mitigate market imperfections, enabling increased investment in innovation and R&D activities. By alleviating high upfront risks, subsidies help firms achieve socially optimal R&D levels, thereby enhancing productivity [13–15]. Second, subsidies improve resource allocation efficiency by channeling resources toward firms with higher productivity potential and correcting market distortions induced by taxation [16,17]. Additionally, subsidies serve as credible signals of firm quality, reducing information asymmetries and enhancing firms’ access to further resources [16].

However, the effectiveness of government subsidies in enhancing TFP appears to be context-dependent. Studies suggest that subsidy impacts are more significant in competitive markets [18], and the beneficial effects often exhibit substantial time lags, potentially producing negligible or negative outcomes in the short run [14,19,20]. Moreover, targeted subsidies, particularly those focused on innovation activities, tend to demonstrate more pronounced positive effects [16,18].

Evidence suggests that the impact of subsidies varies across firm types, notably among small and medium-sized enterprises (SMEs), non-state-owned enterprises (non-SOEs), and technology-intensive firms. Subsidies typically exert stronger positive influences on these entities, enhancing external financing access, innovation capability, and market expansion, thereby resulting in significant productivity gains [14,21–24].

In contrast, some scholars argue subsidies can negatively affect TFP [25]. First, automatic or broadly distributed subsidies may reduce firms’ motivation for innovation [26]. Second, poorly designed subsidy schemes might allocate resources predominantly to low-productivity firms, impeding resource allocation efficiency and overall economic performance [27,28]. Moreover, rent-seeking behaviors during subsidy distribution processes can distort business practices and resource allocation, consequently suppressing TFP [29]. Finally, conflicting social objectives of subsidies, such as environmental protection or job creation, might further complicate TFP goals [30].

In light of the above concerns, given these potential drawbacks, scholars emphasize the importance of careful subsidy policy design, advocating specificity and efficiency to prevent resource misallocation and rent-seeking, ensuring subsidies genuinely stimulate productivity improvements [20,31]. Empirical evidence suggests subsidy policy reforms can shift effects from negative to positive [18,32].

China’s experience with subsidies reflects complex dynamics. Some researchers demonstrate positive effects of subsidies in China’s manufacturing sector through technological progress and resource allocation improvements [33]. However, contradictory findings highlight frequent subsidy allocation to inefficient firms, particularly SOEs, resulting in negative productivity outcomes due to reduced incentives for efficiency and innovation [31,34].

### Government subsidies and the formation of zombie firms

Zombie firms are enterprises unable to independently sustain operations, continuously operating at losses without prospects of regaining profitability, yet remaining active due to external financial support, such as government subsidies, extended bank loans, or other assistance [35]. The existence of these firms has drawn considerable attention due to their significant negative implications for economic efficiency and resource allocation [36,37].

First, zombie firms exhibit persistently low productivity and weak innovation capacity, directly dragging down average productivity at the aggregate level [38–41]. Second, by absorbing substantial resources such as credit, land, and labor through non-market mechanisms, zombie firms crowd out more efficient and innovative firms, stifling innovation, distorting market selection, and ultimately suppressing overall productivity growth [42–45].

The persistence of zombie firms is generally attributed to continuous external financial support. Among the main channels of such support, bank lending [46–48] and government subsidies [40,49] play particularly important roles. However, there remains considerable debate over the role of subsidies in perpetuating zombie firms. Government assistance does not necessarily lead to zombification; in many cases, subsidies may exert positive effects, as discussed earlier, by enhancing firms’ innovation incentives and productivity. Even for low-efficiency or financially distressed firms, government support does not inevitably result in zombification but can instead facilitate restructuring and performance recovery [50]. Therefore, the impact of government subsidies on the formation of zombie firms is context-dependent and varies with firms’ characteristics and the design of subsidy policies.

Extant literature extensively explores the individual impacts of government subsidies on TFP and the persistence of zombie firms. However, few studies integrate these two dimensions to investigate how government subsidies influence TFP via zombie firm formation. Existing research frequently neglects the mediating role of zombie firms in assessing subsidy effectiveness. To fill this gap, this paper systematically examines how government subsidies affect TFP through the formation of zombie firms. Through empirical analysis of the underlying mechanisms and effects, our study contributes to the broader literature on government intervention and productivity dynamics, providing empirical evidence and theoretical insights to support policymakers in designing more effective subsidy strategies to enhance economic efficiency.

### Theoretical analysis

This section constructs a theoretical framework to analyze firm production decisions at both macroeconomic and microeconomic levels. Specifically, it examines the influence of government subsidies on firms’ market exit behavior and elucidates the mediating mechanism through which subsidies affect aggregate TFP. We assume that industry output follows a Constant Elasticity of Substitution (CES) production function aggregating outputs from micro-firms under monopolistic competition, which is formally represented as:


$$\begin{array}{c} \text{Y}={\left(\sum {\mathrm{\backslash nolimits}}_{\text{i}=\text{1}}^{{\text{M}}_{\text{s}}}{\text{Y}}_{\text{i}}^{\frac{\sigma -\text{1}}{\sigma }}\right)}^{\frac{\sigma }{\sigma -\text{1}}} \end{array}$$ (1)

In equation (1), Y and ${\text{Y}}_{\text{i}}$ represent the output of industry S and the output of micro-firm i, respectively. The term ${\text{M}}_{\text{s}}$ denotes the number of micro-firms, and σ is the elasticity of substitution between outputs of micro-firms. The output and income functions of micro-firm i can be derived as:


$$\begin{array}{c} {\text{Y}}_{\text{i}}\;=\;{\text{Y}\left({\text{P}}_{\text{i}}/\text{P}\right)}^{-\sigma } \end{array}$$ (2)

$$\begin{array}{c} {\text{R}}_{\text{i}}\;=\;{\text{R}\left({\text{P}}_{\text{i}}/\text{P}\right)}^{\text{1}-\sigma } \end{array}$$ (3)

In equation (2), P and ${\text{P}}_{\text{i}}$ represent the prices of industry S and micro-firm i, respectively. In equation (3), R and ${\text{R}}_{\text{i}}$ denote incomes for industry S and the micro-firm i, respectively.

Suppose the industry comprises two categories of micro-firms: the first includes government-subsidized firms (denoted by subscript s) with a subsidy rate ϕ, and the second includes unsubsidized firms (denoted by subscript n). Each firm enters the market and randomly draws its productivity level φ. Following Melitz (2003), we assume that both types of firms have productivity levels that are independently and identically distributed according to a Pareto cumulative distribution function (CDF). The total production cost for micro-firm i is represented by ${\text{C}}_{\text{i}}=f+\frac{{\text{Y}}_{\text{i}}}{\varphi }$, where f indicates fixed costs, assumed uniform across all firms.

We calculate the profit functions for subsidized and unsubsidized firms as follows:


$$\begin{array}{c} \pi_{\text{n}}\;=\;{\text{P}}_{\text{i}}{\text{Y}}_{\text{i}}-\left(\text{f}+{\text{Y}}_{\text{i}}/\varphi \right) \end{array}$$ (4)

$$\pi_{\text{s}}=\left(\text{1}+\phi \right){\text{P}}_{\text{i}}{\text{Y}}_{\text{i}}-\left(\text{f}+{\text{Y}}_{\text{i}}/\varphi \right)$$ (5)

Utilizing first-order conditions for profit maximization, we derive the equilibrium output prices for unsubsidized and subsidized firms:


$${\text{P}}_{\text{n}}=\frac{\sigma }{\left(\sigma -\text{1}\right)\varphi }$$ (6)

$${\text{P}}_{\text{s}}=\frac{\sigma }{\left(\sigma -\text{1}\right)\left(\text{1}+\phi \right)\varphi }$$ (7)

When firms reach the zero-profit condition, they approach the critical threshold for market exit. At this critical point, revenues for unsubsidized and subsidized firms, respectively, can be expressed as:


$${\text{R}}_{\text{n}}\left(\varphi_{\text{n}}^{*}\right)=\sigma \text{f}$$ (8)

$${\text{R}}_{\text{s}}\left(\varphi_{\text{s}}^{*}\right)=\frac{\sigma \text{f}}{\text{1}+\phi }$$ (9)

where $\varphi_{n}^{*}$ and $\varphi_{s}^{*}$ denote the cutoff productivity levels of unsubsidized and subsidized firms, respectively. Additionally, firm revenues at the critical exit threshold can be expressed alternatively as:


$${\text{R}}_{\text{n}}\left(\varphi_{\text{n}}^{*}\right)={\text{R}\left[\frac{\sigma }{\left(\sigma -\text{1}\right)\varphi_{\text{n}}^{*}\text{P}}\right]}^{\text{1}-\sigma }$$ (10)

$${\text{R}}_{\text{s}}\left(\varphi_{\text{s}}^{*}\right)={\text{R}\left[\frac{\sigma }{\left(\sigma -\text{1}\right)\left(\text{1}+\phi \right)\varphi_{\text{s}}^{*}\text{P}}\right]}^{\text{1}-\sigma }$$ (11)

Combining equations (10) and (11), we obtain the relationship between the critical productivity levels ($\varphi_{n}^{*},\varphi_{s}^{*}$) of unsubsidized and subsidized firms:


$$\frac{\varphi_{\text{s}}^{*}}{\varphi_{\text{n}}^{*}}={\left(\frac{\text{1}}{\text{1}+\phi }\right)}^{\frac{\sigma }{\sigma -\text{1}}}$$ (12)

which shows that subsidies lower the survival cutoff. Since the elasticity of substitution typically exceeds unity [51], subsidized firms face a lower productivity threshold and enjoy enhanced competitiveness. This leads to the following key theoretical insights:

First, since ${\varphi_{s}^{*}<\varphi }_{\text{n}}^{*}$, government subsidies reduce the minimum productivity required for subsidized firms to remain in the market, thereby decreasing their exit probability.

**Fig 1:**
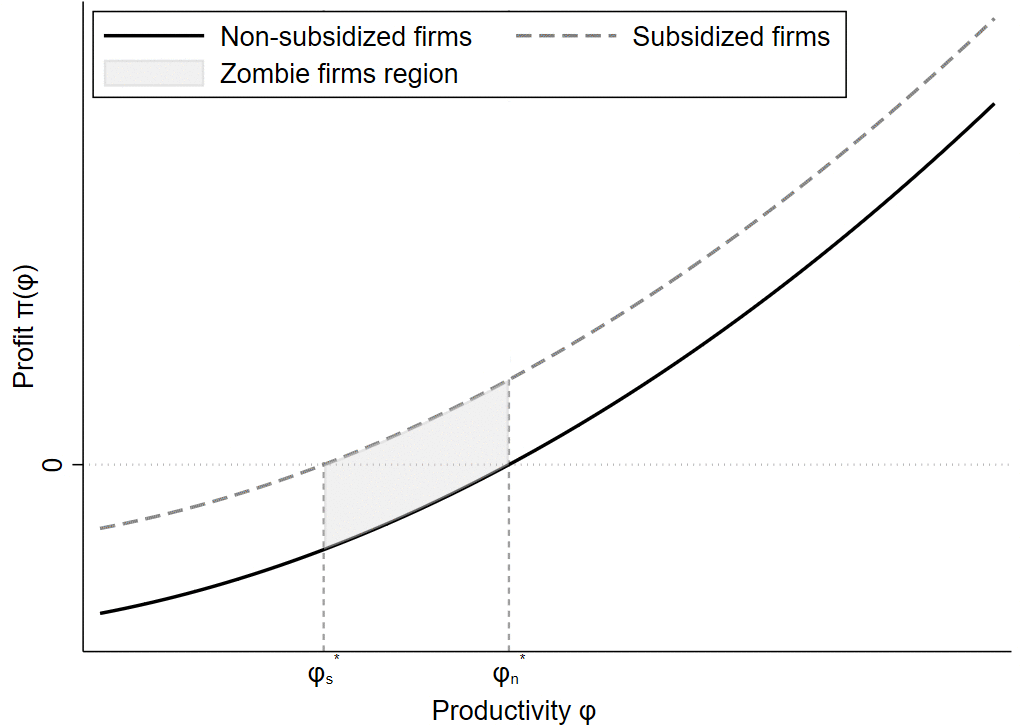
Selection Mechanism with Subsidies and Zombie Firms.

Second, low-productivity firms, with TFP levels between $\varphi_{\text{s}}^{*}$ and $\varphi_{\text{n}}^{*}$, which would otherwise exit in the absence of subsidies—tend to survive due to government support, becoming potential zombie firms. As illustrated clearly in Fig 1, the shaded region between the two cutoffs visually captures this mechanism: firms that lie below the non-subsidized exit threshold but above the subsidized exit threshold are sustained only through subsidies, forming the pool of zombie firms.“

Finally, when a subset of firms receive government subsidies, the corrected CES aggregation—following Dixit and Stiglitz (1977) and Melitz (2003)—implies that the aggregate TFP of the industry, in which subsidized and unsubsidized firms coexist, can be expressed as: $\overset{―}{\varphi }\left(\varphi_{n}^{*},\varphi_{s}^{*}\right)=\left(\text{1}-\theta \right)\;\overset{―}{\varphi }{\left(\varphi_{n}^{*}\right)}^{\sigma -\text{1}}+\theta \;\overset{―}{\varphi }{\left(\varphi_{s}^{*}\right)}^{\sigma -\text{1}},\;\theta \in \left(\text{0},\text{1}\right)$. where θ is the share of subsidized survivors in total survivors, and $\overset{―}{\varphi }\left(\varphi_{i}^{*}\mathrm{\backslash rightleft}(i=n,\;s\right)$ denotes the average productivity of firms that survive above the cutoff productivity level $\varphi_{i}^{*}.$ Since subsidies reduce the cutoff productivity, $\varphi_{s}^{*}<\varphi_{n}^{*}$, and $\overset{―}{\varphi }(\cdot )\;$is strictly increasing in the cutoff, it follows that $\overset{―}{\varphi }\left(\varphi_{s}^{*}\right)<\overset{―}{\varphi }\left(\varphi_{n}^{*}\right)$. Hence, the aggregate industry productivity under partial subsidies satisfies: $\overset{―}{\varphi }\left(\varphi_{n}^{*},\varphi_{s}^{*}\right)<\overset{―}{\varphi }\left(\varphi_{n}^{*}\right)$. This result indicates that government subsidies, by allowing low-productivity firms to remain in the market, ultimately reduce the overall productivity of the industry.

This mechanism reflects a form of resource misallocation different from that described by Hsieh and Klenow (2009). While their framework emphasizes misallocation arising from factor price distortions, the present analysis highlights an exit-barrier mechanism, in which subsidies distort firm selection by allowing inefficient firms to remain in the market.

### Empirical models setting and data description

Based on the theoretical framework above, this section presents empirical models, defines variables, and describes sample data.

### Setting of empirical models

To empirically test the theoretical relationship between government subsidies and firm exit at the firm level, we construct the following econometric model:


$${\text{Ex}}_{\text{it}}=\alpha_{\text{0}}+\alpha_{\text{1}}\text{Sub}{\text{d}}_{\text{it}}+{\beta \text{X}}_{\text{it}}+\sum \nu_{\text{n}}\text{in}{\text{d}}_{\text{n}}+\sum \lambda_{\text{t}}\text{yea}{\text{r}}_{\text{t}}+\epsilon_{\text{it}}$$ (13)

where $Ex_{it}$ denotes a dummy variable indicating whether the firm i exits the market in year t. $Subd_{it}$ represents whether the firm receives government subsidies. $X_{it}$ is a vector of control variables, including firm age ($Age_{it}$), ownership type ($OwnType_{it}$), regional location ($Reg_{it}$), proportion of R&D personnel ($RDPers_{it}$), proportion of R&D investment ($RDInv_{it}$), asset–liability ratio ($ALRatio_{it}$), and return on assets ($ROA_{it}$). $ind_{m}$ and $year_{t}$ denote industry and year fixed effects, respectively, and $\epsilon_{it}$ is the error term.

Similarly, to examine the effect of subsidies on the formation of zombie firms at the firm level, we estimate:


$${\text{Zo}}_{\text{it}}=\alpha_{\text{0}}+\alpha_{\text{1}}\text{Sub}{\text{d}}_{\text{it}}+{\beta \text{X}}_{\text{it}}+\sum \nu_{\text{n}}\text{in}{\text{d}}_{\text{n}}+\sum \lambda_{\text{t}}\text{yea}{\text{r}}_{\text{t}}+\epsilon_{\text{it}}$$ (14)

In equation (14), $Zo_{it}$ denotes a dummy variable indicating whether the firm is a zombie firm. All other variables retain the definitions specified in equation (13).

To empirically test the mediating effect of zombie firm formation in the relationship between government subsidies and aggregate TFP at the industry-province-year level, we adopt the stepwise regression approach [52]. Specifically, the following sequential regression equations are specified:


$${\text{TFP}}_{\text{jpt}}=\alpha_{\text{10}}+\alpha_{\text{11}}{\text{SubInt}}_{\text{jpt}}+\beta_{\text{1}{\text{X}}_{\text{jpt}}}+\lambda_{\text{t1}}+\epsilon_{\text{1},\text{jpt}}$$ (15)

$${\text{ZoShare}}_{\text{jpt}}=\alpha_{\text{20}}+\alpha_{\text{21}}{\text{SubInt}}_{\text{jpt}}+\beta_{\text{2}}{\text{X}}_{\text{jpt}}+\lambda_{\text{t2}}+\epsilon_{\text{2},\text{jpt}}$$ (16)

$${\text{TFP}}_{\text{jpt}}=\alpha_{\text{30}}+\alpha_{\text{31}}{\text{SubInt}}_{\text{jpt}}+\alpha_{\text{32}}{\text{ZoShare}}_{\text{jpt}}+\beta_{\text{3}}{\text{X}}_{\text{jpt}}+\lambda_{\text{t3}}+\epsilon_{\text{3},\text{jpt}}$$ (17)

where $TFP_{jpt}$ denotes the aggregate TFP for industry j in province p and year t. $SubInt_{jpt}$ represents subsidy intensity. $ZoS\text{h}are_{jpt}$ denotes the share of zombie firms. $X_{jpt}$ is a vector of control variables, including the R&D personnel ratio ($RDPers_{jpt}$), R&D investment ratio ($RDInv_{jpt}$), asset–liability ratio ($ALRatio_{jpt}$), and return on assets ($ROA_{jpt}$). $\lambda_{t}$ captures year fixed effects, and $\epsilon_{jpt}$ is the idiosyncratic error term.

The coefficient $\alpha_{\text{11}}$ in equation (15) measures the total effect of government subsidies on aggregate productivity. The coefficient $\alpha_{\text{21}}$ in equation (16) captures the effect of subsidies on the formation of zombie firms. The coefficient $\alpha_{\text{31}}$in equation (17) measures the direct effect of subsidies on productivity, while $\alpha_{\text{32}}$ reflects the effect of zombie-firm prevalence on productivity. The indirect (mediating) effect of government subsidies on productivity through zombie firms is identified as: $IE=\alpha_{\text{21}}\times \alpha_{\text{32}}$.

### Data description

This study employs an unbalanced panel dataset of Chinese A-share listed firms covering the period from 2007 to 2022. The empirical analysis is conducted at two levels, the micro (firm) level and the meso (industry-province-year) level, corresponding to the distinct hypotheses derived from the theoretical framework.

To test the micro-level mechanisms proposed in Hypotheses 1 and 2, firm-level regressions are performed to examine the effects of government subsidies on firm exit and zombie-firm formation. Financial firms and observations with missing key variables are excluded to ensure sample consistency and robustness.

Exit (Ex): Denotes a dummy variable equal to 1 if a firm is delisted, suspended, or placed under special treatment (“ST”) status in the A-share market, and 0 otherwise. This variable captures capital-market exit, consistent with prior studies using listed-firm samples in China. Although it does not perfectly represent economic shutdown or bankruptcy, it serves as a practical proxy for firm exit given the limitations of publicly available data.

Zombie Firms (Zo): Identified using the FN–CHK method proposed by Fukuda and Nakamura (2011) [53]. A firm is classified as a zombie if its earnings before interest and taxes (EBIT), after deducting government subsidies, are insufficient to cover interest expenses (interest coverage ratio < 1). To distinguish genuine zombie firms from newly established or temporarily distressed firms, additional criteria are applied: (1) the firm has operated for more than three years; (2) the debt ratio in yeart exceeds 50%; and (3) total liabilities in yeart are greater than those in yeart−1.

Total factor productivity (TFP): Estimated using the Olley–Pakes (OP) method [54], which effectively corrects both simultaneity and selection biases inherent in production function estimation. The simultaneity problem arises because firms adjust their input choices based on their current productivity, causing inputs to be endogenous. OP alleviates this bias by assuming that firm investment is a strictly monotonic function of productivity, allowing investment to serve as a proxy for unobserved productivity shocks. In addition, OP mitigates selection bias by explicitly modeling firm exit, recognizing that low-productivity firms are more likely to leave the market, which would otherwise lead to survivor-based estimation bias. Following this approach, we estimate a Cobb–Douglas production function of the form:


$${\text{y}}_{\text{it}}=\;\alpha +\beta_{\text{K}}{\text{k}}_{\text{it}}+\beta_{\text{L}}{\text{l}}_{\text{it}}+\beta_{\text{A}}\text{Ag}\text{e}+\beta_{\text{O}}\text{OwnTyp}{\text{e}}_{\text{it}}+\beta_{\text{e}}{\text{Ex}}_{\text{it}}$$

$$\begin{array}{c} +\sum {\mathrm{\backslash nolimits}}_{m}\lambda_{m}\text{Yea}{\text{r}}_{\text{m}}+\sum {\mathrm{\backslash nolimits}}_{n}\lambda_{n}\text{In}{\text{d}}_{\text{n}}+\varepsilon_{it} \end{array}$$ (18)

where $y_{it}$ denotes the logarithm of operating revenue, $k_{it}$ the logarithm of net fixed assets, and $l_{it}$ the logarithm of the number of employees. Firm age ${Age}_{it}$ and ownership type ${OwnType}_{it}$enter the production function as additional control variables, while Year and Ind dummies account for macroeconomic and structural heterogeneity. Firm-level TFP is then computed as the OP-estimated productivity term ${\hat{\omega }}_{it}$.

Subsidy (Subd): Given the widespread prevalence of subsidies among listed firms, subsidy intensity is measured as the ratio of total subsidies to total assets. A binary indicator is further constructed, coded as 1 if a firm’s subsidy intensity is above the sample median (subsidized) and 0 otherwise (unsubsidized).

Firm age (Age): Calculated as the current year minus the firm’s founding year.

Ownership type (OwnType): Equals 1 for state-owned enterprises (SOEs) and 0 for non-state-owned enterprises (non-SOEs).

Regional location (Reg): Equals 1 for firms located in central or western regions and 0 for firms in eastern regions.

Proportion of R&D personnel (RDPers): The ratio of R&D personnel to total employees.

Proportion of R&D investment (RDInv): The ratio of R&D expenditure to operating revenue.

Asset-liability ratio (ALRatio): Total liabilities divided by total assets.

Return on assets (ROA): Net income divided by average total assets.

The summary statistics for the micro-level variables are presented in Table 1.

**Table 1:** Descriptive statistics for firm-level variables.

Variable	N	Mean	Std. Dev.	Min	Max
Ex	34,048	0.031	0.173	0	1
Zo	34,048	0.071	0.256	0	1
TFP	34,045	6.154	1.211	0.003	11.205
Subd	34,048	0.485	0.5	0	1
Age	34,048	12.234	7.068	2	33
OwnType	34,048	0.405	0.491	0	1
Reg	34,048	0.305	0.46	0	1
RDPers	34,048	0.094	0.132	0	0.945
RDInv	34,048	0.035	0.046	0	0.263
ALRatio	34,048	0.453	0.207	0.065	0.967
ROA	33,796	0.044	0.175	−1.108	0.335

Note: The number of observations varies across variables due to missing data.

To examine the meso-level effects proposed in Hypothesis 3, a balanced panel of industry–province–year observations is constructed using firm-level weights based on firm count or total assets. The analysis investigates how government subsidies and the prevalence of zombie firms jointly affect aggregate TFP at the industry–province level.

Aggregate TFP (TFP): The weighted average total factor productivity of firms within each industry–province–year cell, adjusted using CES aggregation following Melitz (2003) [55].

Subsidy Intensity (SubInt): The average ratio of total subsidies to total assets within each cell.

Zombie Share (ZoShare): The proportion of zombie firms among all firms within each cell.

R&D Personnel Share (RDPers) and R&D Investment Share (RDInv): The aggregated averages of the ratios of R&D personnel and R&D expenditure, respectively, within each cell.

Asset-Liability Ratio ALRatio) and ROA: The aggregated averages of firm-level financial indicators within each cell, capturing financial health and profitability.

The summary statistics for the meso-level variables are presented in Table 2.

**Table 2:** Descriptive Statistics for Firm-Level Variables.

Variable	N	Mean	Std. Dev.	Min	Max
TFP	11,696	6.733	1.193	1.901	11.145
SubInt	11,696	0.005	0.009	0	0.285
ZoShare	11,696	0.076	0.238	0	1
RDPers	11,696	0.057	0.096	0	0.890
RDInv	11,696	0.020	0.030	0	0.263
ALRatio	11,696	0.499	0.185	0.065	0.967
ROA	11,696	0.053	0.136	−0.704	0.307

## Empirical results and discussions

This section presents the empirical analysis based on the econometric models described previously.

### The effect of government subsidies on firms’ exit

We first conducted a preliminary analysis using a t-test to examine the differences in exit rates between subsidized and non-subsidized firms. Table 3 presents evidence that non-subsidized firms had a significantly higher exit rate (3.73%) compared to subsidized firms (2.41%), resulting in a mean difference of −1.38%, with a highly significant t-statistic of −7.05 (p < 0.01).

**Table 3:** Results of T-tests for 𝐄𝐱 Variable Before and After Matching.

Sample	Subsidized	Non-Subsidized	Difference	S.E.	T-stat
Unmatched	0.0241	0.0373	−0.0132	0.0019	−7.05***
Matched	0.0257	0.0357	−0.0100	0.0022	−4.53***

Note: *** p < 0.01, ** p < 0.05, * p < 0.10.

However, as government subsidies are unlikely to be randomly assigned—given that subsidized firms may differ systematically from non-subsidized firms—potential selection bias may arise, thereby confounding the relationship between subsidies and firm exit. To address this concern, we utilized propensity score matching (PSM), a method that balances observable firm characteristics and creates a more comparable sample between the two groups.

After matching, the differences in exit rates remained statistically significant, albeit slightly reduced. Specifically, the matched exit rate was 2.57% for subsidized firms versus 3.57% for non-subsidized firms, resulting in a mean difference of −1.00% and a significant t-statistic of −4.53 (p < 0.01). These findings confirm that government subsidies continue to significantly reduce firms’ likelihood of exiting the market, even after controlling for observable characteristics.

Next, following the empirical specification outlined in equation (13), we examined the relationship between government subsidies and firm exit using a Probit model, suitable for the binary nature of the dependent variable. Additionally, we accounted for potential reverse causality between government subsidies and firm exit decisions: firms approaching market exit conditions may be targeted for subsidies due to social stability or employment considerations, leading to endogeneity bias if left unaddressed.

To mitigate this concern, we employed an instrumental variable (IV) Probit approach, using firms’ export status as an instrumental variable. Export status meets the relevance criterion—exporting firms are more likely to receive subsidies—and satisfies the exclusion restriction since exporting itself does not directly affect a firm’s exit probability independently of subsidy receipt.

The IV Probit estimation results validate the effectiveness of the instrumental variable, with AR and Wald tests yielding significant results (p < 0.01), indicating no issues with weak instruments and confirming that export status strongly predicts subsidy allocation.

Table 4 presents evidence that government subsidies are significantly associated with a lower probability of firm exit, based on results from both the standard Probit and IV Probit models. The IV Probit estimation confirms that, after addressing potential endogeneity, the negative association between subsidies and exit remains robust and statistically significant. This finding indicates that firms receiving government support are less likely to exit the market, possibly due to enhanced financial stability and competitiveness.

**Table 4:** Estimated Results of the Impact of Government Subsidies on Firms’ Exit.

Methods	Probit	IVProbit	
Estimation stage and variables	Ex	First stage regression Subd	Second stage regression Ex
Subd	−0.124*** (0.0341)		−4.127*** (1.071)
Expd		0.0396*** (0.00579)	
Age	0.0189*** (0.00264)	−0.00310*** (0.000453)	0.00637 (0.00462)
OwnType	−0.781*** (0.0409)	−0.00178 (0.00642)	−0.800*** (0.0487)
Reg	0.184*** (0.0340)	0.0181*** (0.00594)	0.239*** (0.0439)
RDPers	0.151 (0.188)	0.0637** (0.0311)	0.422* (0.237)
RDInv	−1.955*** (0.559)	1.872*** (0.0852)	5.601*** (2.123)
ALRatio	0.753*** (0.0903)	0.0280* (0.0154)	0.901*** (0.117)
ROA	−0.839*** (0.0635)	0.126*** (0.0154)	−0.322* (0.165)
cons	−2.482*** (0.371)	0.342*** (0.0371)	−1.109** (0.543)
Year	YES	YES	YES
Ind	YES	YES	YES
AR			21.21***
Wald			14.84***
N	31443	31443	31443

Notes: *** p < 0.01, ** p < 0.05, * p < 0.10; Standard errors is in parentheses; AR and Wald respectively represent the P-values for the AR and Wald Tests; N represents sample size.

Additionally, coefficient estimates for control variables are consistent with theoretical expectations, further reinforcing the robustness of our empirical findings.

### The effect of government subsidies on the formation of zombie firms

The empirical findings discussed above demonstrate that government subsidies lower firms’ likelihood of exiting the market. However, subsidies may have a dual effect: they can enhance the competitiveness of productive firms, but they may also prolong the survival of inefficient firms that would otherwise exit the market. This dual role highlights the importance of further examining whether government subsidies promote the formation of zombie firms.

**Fig 2:**
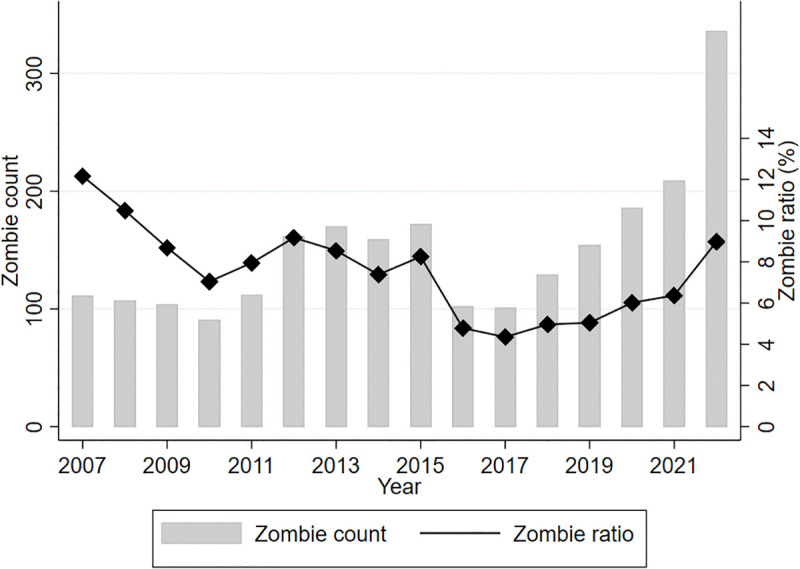
Trends in Zombie Firms: Count and Proportion (2007-2022).

Using the zombie firm identification criteria detailed previously, we computed the annual proportion and total number of zombie firms, as illustrated in Fig 2. Our findings indicate an average proportion of zombie firms of approximately 8.05% throughout the sample period, underscoring their persistent presence within the economy. Notable fluctuations were observed during different economic periods. Specifically, the proportion peaked between 2007 and 2009—reaching its highest level of 12.16% in 2007—likely due to the global financial crisis. Between 2010 and 2015, the proportion remained relatively high, ranging from 7.04% to 8.25%, possibly reflecting accommodative monetary policies that prolonged the lifespan of inefficient firms. Subsequently, during the period from 2016 to 2019, the proportion sharply decreased, reaching its lowest level of 4.35% in 2017, presumably resulting from supply-side structural reforms designed to enhance economic efficiency. Conversely, from 2020 to 2022, the proportion rose significantly again—from 6.01% in 2020 to 8.98% in 2022—likely reflecting economic disruptions caused by the COVID-19 pandemic. The absolute number of zombie firms has also steadily increased since 2019, underscoring that the problem has intensified, particularly following the pandemic’s onset.

These findings highlight the persistent and cyclical nature of the zombie firm phenomenon. Despite temporary improvements associated with structural reforms, zombie firms remain an enduring challenge that continues to hinder resource reallocation and economic efficiency, underscoring the need for targeted policy responses.

To assess the impact of subsidies on zombie firm formation more precisely, we performed a t-test comparing the prevalence of zombie firms between subsidized and non-subsidized groups. Table 5 presents the results, showing that prior to matching, subsidized firms exhibited a higher average proportion of zombie status (8.10%) compared to non-subsidized firms (6.10%), with a statistically significant difference of 1.94% (p < 0.01). After propensity score matching, the average proportion of zombie firms remained almost unchanged for both groups, 8.10% for subsidized firms and 6.20% for non-subsidized firms, yielding a consistent and statistically significant difference of 1.84% (p < 0.01). These findings indicate that the positive association between government subsidies and the likelihood of zombification persists even after controlling for observable firm characteristics.

**Table 5:** Results of T-tests for ZO variable before and after matching.

Sample	Subsidized	Non -Subsidized	Difference	S.E.	T-stat
Unmatched	0.081	0.061	0.0194	0.0028	7.00***
Matched	0.081	0.062	0.0184	0.0028	6.48***

Note: *** p < 0.01, ** p < 0.05, * p < 0.10.

Subsequently, employing empirical model (14), we analyzed the relationship between government subsidies and zombie firm formation using a Probit model, consistent with the binary nature of the dependent variable. Given potential endogeneity arising from reverse causality—where the likelihood of becoming a zombie firm may influence subsidy allocation—we applied an instrumental variable (IV) Probit approach, with firms’ export status serving as the instrument. This variable satisfies the relevance criterion—exporting firms are more likely to receive subsidies—and the exclusion restriction, as export activity itself is unlikely to directly affect zombification. The instrument’s validity is confirmed by significant AR and Wald tests (both p < 0.01), indicating its robustness in addressing endogeneity concerns.

Table 6 presents the IV Probit estimation results, showing that government subsidies exert a statistically significant positive effect on the probability of zombie firm formation. This finding supports the hypothesis that subsidies, by sustaining low-productivity firms that would otherwise exit, contribute directly to the persistence and proliferation of zombie firms.

**Table 6:** Estimated Results of the Impact of Government Subsidies on the Formation of Zombie Firms.

Methods	Probit	IVProbit	
Estimation stage and variables	Zo	First stage regression Subd	Second stage regression Zo
Subd	0.244*** (0.0260)		1.063*** (0.296)
Expd		0.0823*** (0.00535)	
Age	0.00431** (0.00214)	−0.00410*** (0.000409)	−0.00139 (0.00227)
OwnType	0.0517* (0.0294)	−0.0159*** (0.00600)	0.160*** (0.0279)
Reg	0.0217 (0.0267)	0.0243*** (0.00574)	0.0519** (0.0256)
RDPers	−0.0577 (0.151)	0.144*** (0.0261)	−0.996*** (0.136)
RDInv	2.594*** (0.363)	2.515*** (0.0783)	0.378 (0.836)
ALRatio	1.459*** (0.0737)	−0.116*** (0.0143)	1.429*** (0.0749)
ROA	−1.908*** (0.0516)	0.0631*** (0.0152)	−1.936*** (0.0542)
cons	−1.609*** (0.137)	0.440*** (0.00922)	−2.732*** (0.149)
Year	YES	YES	YES
Ind	YES	YES	YES
AR			13.21***
Wald			12.86***
N	33710	33710	33796

Note: *** p < 0.01, ** p < 0.05, * p < 0.10; Standard errors is in parentheses; AR and Wald respectively represent the P-values for the AR and Wald Tests; N represents sample size.

Existing literature indicates that SOEs typically exhibit lower efficiency than non-SOEs due to government protection, inefficient resource allocation, and limited market competition [56,57]. Similarly, firms located in China’s less-developed Central and Western regions are generally less efficient than those in the more economically advanced Eastern region [58–60]. Based on these observations, we hypothesize that SOEs and firms located in Central and Western China are more likely to become zombie firms.

To test this hypothesis, we conducted t-tests comparing the prevalence of zombie status between SOEs and non-SOEs, as well as between firms located in Central and Western regions versus the Eastern region. Table 7 summarizes the results, indicating that both ownership structure and geographic location significantly affect the likelihood of firms becoming zombies. Specifically, SOEs exhibit a higher zombie incidence (8.61%) than non-SOEs (6.01%), resulting in a statistically significant difference of 2.60 percentage points (p < 0.01). Similarly, firms in Central and Western regions display a higher average probability of zombification (8.47%) compared to those in the Eastern region (6.44%), with a significant difference of 2.02 percentage points (p < 0.01).

**Table 7:** T-Test Results for the Probability of Becoming Zombie Firms: Ownership Type and Regional Comparisons.

Sample	Mean (Group 0)	Mean (Group 1)	Difference	S.E.	T-stat
State-owned vs. Non-state-owned	0.0861	0.0601	0.0260	0.0029	8.91***
Central & Western vs. Eastern	0.0847	0.0644	0.0202	0.0032	6.40***

Notes: *** p < 0.01, ** p < 0.05, * p < 0.10.

These findings suggest that state ownership and regional economic development not only influence firms’ propensity to become zombies but may also moderate the broader impact of government subsidies on aggregate productivity through their effect on zombie firm formation.

Table 8 presents the results of further empirical analysis based on Probit estimations, which reveal notable heterogeneity in the effects of government subsidies on zombie firm formation across ownership and regional dimensions. In particular, the positive association between subsidies and zombification is significantly stronger for SOEs and for firms located in Central and Western regions.

**Table 8:** Estimated Results of the Comparison Between Ownership Type and Regional Differences.

Methods	Probit			
Variables	State-owned	Non-state-owned	Central&Western	Eastern
Subd	0.364*** (0.0391)	0.131*** (0.0356)	0.336*** (0.0449)	0.190*** (0.0325)
Expd	0.012*** (0.00366)	0.00086 (0.00289)	0.00722* (0.00389)	0.00325 (0.00265)
Age			0.0917* (0.0492)	0.0117 (0.0379)
OwnType	0.0317 (0.0388)	−0.0010 (0.0390)		
Reg	0.149 (0.277)	−0.122 (0.182)	−0.254 (0.322)	−0.0105 (0.174)
RDPers	1.709** (0.762)	2.947*** (0.421)	3.199*** (0.717)	2.431*** (0.428)
RDInv	1.495*** (0.115)	1.439*** (0.0993)	1.264*** (0.129)	1.555*** (0.0915)
ALRatio	−2.139*** (0.0851)	−1.766*** (0.0668)	−1.794*** (0.0875)	−1.991*** (0.0650)
ROA	−1.654*** (0.170)	−1.675*** (0.271)	−1.714*** (0.188)	−1.186*** (0.216)
Year	YES	YES	YES	YES
Ind	YES	YES	YES	YES
N	13443	19949	10007	23282

Note: *** p < 0.01, ** p < 0.05, * p < 0.10; Standard errors is in parentheses; N represents sample size.

### The Mediating Role of Zombie Firms in the Relationship Between Government Subsidies and Aggregate TFP

Prior to formally testing the mediation hypothesis, we conducted a preliminary t-test comparing TFP between zombie and non-zombie firms. Table 9 reports significant productivity differences between the two groups. In the unmatched sample, zombie firms exhibited an average TFP of 6.03, which was significantly lower than the 6.17 observed for non-zombie firms, yielding a mean difference of −0.14 (p < 0.01). This result highlights the substantially lower productivity levels characterizing zombie firms.

**Table 9:** T-Test Results for the Difference in TFP Between Zombie and Non-Zombie Firms.

Sample	Zo	Non-Zo	Difference	S.E.	T-stat
Unmatched	6.0285	6.1674	−0.1389	0.0259	−5.37***
Matched	6.0281	6.4491	−0.4210	0.0352	−11.94***

Notes: *** p < 0.01, ** p < 0.05, * p < 0.10.

After applying propensity score matching to control for potential confounding factors affecting firm productivity, the productivity gap remained robust. In the matched sample, zombie firms had an average TFP of 6.03, compared with 6.45 for non-zombie firms, resulting in a larger and still highly significant difference of −0.42 (p < 0.01). These findings provide preliminary evidence consistent with the proposed mediating mechanism.

**Fig 3:**
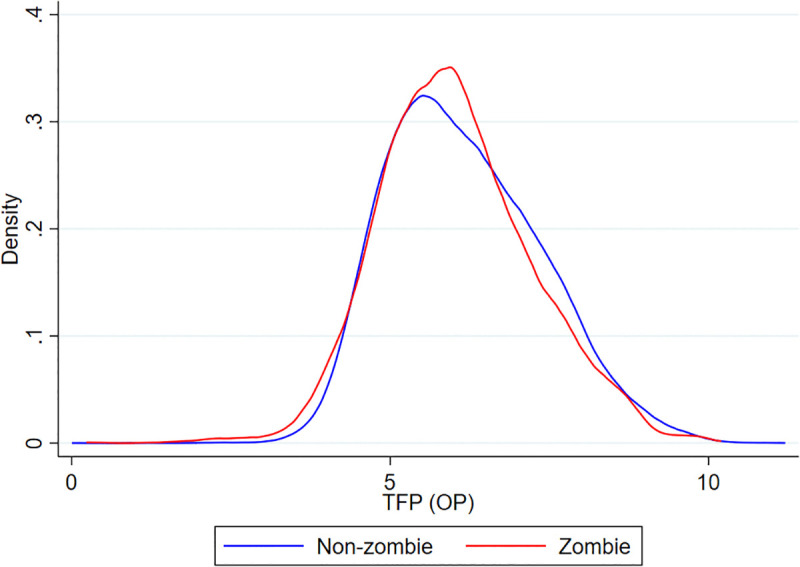
TFP Distribution: Zombie vs Non-zombie.

To complement the mean-difference results in Table 9, we further provide a graphical comparison of the TFP distributions of zombie and non-zombie firms. Fig 3 plots the kernel density estimates of log TFP for both groups. The figure shows that zombie firms are disproportionately concentrated in the lower portion of the productivity distribution and exhibit a much shorter right tail, whereas non-zombie firms display more high-productivity observations. Although the two curves overlap in the middle range, the overall pattern clearly indicates that zombie firms are underrepresented among high-productivity firms and overrepresented in the low-productivity segment. This distributional evidence reinforces the results in Table 9 and supports the view that zombie firms tend to operate with systematically lower productivity.

Based on the preceding empirical analyses, we employed the stepwise mediation testing procedure outlined in equations (15)–(17) to assess whether the presence of zombie firms mediates the relationship between government subsidies and aggregate TFP. Table 10 presents the results from the sequential regression models, showing statistically significant coefficients for the key variables of interest and providing evidence of consistent total, direct, and indirect effects of subsidies on aggregate TFP.

**Table 10:** Estimated Results of the Mediating Role of Zombie Firms in the Relationship Between Government Subsidies and Aggregate TFP.

Variables	TFP	ZoShare	TFP
SubInt	−10.68*** (1.105)	3.200*** (0.214)	−10.05*** (1.115)
ZoShare			−0.197*** (0.0477)
RDPers	2.745*** (0.142)	−0.0941*** (0.0275)	2.726*** (0.142)
RDInv	−6.272*** (0.473)	0.0391 (0.0917)	−6.264*** (0.473)
ALRatio	1.917*** (0.0581)	0.165*** (0.0113)	1.949*** (0.0586)
ROA	1.538*** (0.0769)	−0.662*** (0.0149)	1.408*** (0.0831)
Cons	5.725*** (0.0347)	0.0163** (0.00673)	5.729*** (0.0347)
Year	YES	YES	YES
N	11696	11696	11696
R-squared	0.141	0.191	0.142

Note: *** p < 0.01, ** p < 0.05, * p < 0.10; Standard errors is in parentheses; N represents sample size.

Specifically, the first-stage regression indicates a significantly negative total effect of government subsidies on aggregate TFP, with a coefficient of −10.68. In the second stage, subsidies exert a significantly positive effect on the prevalence of zombie firms, with a coefficient of 3.20. In the third stage, the zombie share has a significantly negative effect on aggregate TFP, with a coefficient of −0.20. Taken together, these results confirm a mediating mechanism whereby government subsidies indirectly reduce aggregate productivity by increasing the prevalence of zombie firms.

Consistent with the preceding t-test results, Table 11 presents the heterogeneous mediation effects of zombie firms across ownership structures. The findings reveal that the mediating effect of zombie firms in the relationship between government subsidies and aggregate TFP is significantly stronger in SOE-dominated industries. This pattern arises from two reinforcing mechanisms. First, government subsidies are more likely to induce zombie firm formation in SOE sectors, where soft budget constraints and limited market competition effectively reduce the TFP threshold required for firm survival, thereby allowing inefficient firms to persist. Second, zombie firms in state-owned industries tend to exhibit markedly lower productivity than the overall firm average, meaning that their presence exerts a stronger downward pull on aggregate TFP. Consequently, industries with a higher SOE share experience a stronger indirect negative impact of subsidies on productivity through the zombie firm channel.

**Table 11:** Ownership Heterogeneity of the Mediating Effects of Zombie Firms in the Relationship Between Government Subsidies and Aggregate TFP.

Variables	Low-SOE			High-SOE		
	TFP	ZoShare	TFP	TFP	ZoShare	TFP
SubInt	-7.782*** (1.716)	1.766*** (0.316)	−9.023*** (1.815)	−8.242*** (1.212)	3.843*** (0.292)	−10.92*** (1.399)
ZoShare			−0.167** (0.0748)			−0.206** (0.0617)
RDPers	0.702*** (0.184)	−0.0208 (0.0340)	2.330*** (0.171)	0.643*** (0.245)	−0.00926 (0.0591)	3.651*** (0.248)
RDInv	-5.912*** (0.558)	0.0974 (0.103)	−5.495*** (0.569)	−7.087*** (0.800)	−0.0344 (0.193)	−4.402*** (0.881)
ALRatio	2.139*** (0.0797)	0.115*** (0.0147)	2.127*** (0.0846)	1.734*** (0.0707)	0.189*** (0.0170)	1.607*** (0.0813)
ROA	1.783*** (0.101)	−0.519*** (0.0186)	1.408*** (0.112)	1.888*** (0.0973)	−0.826*** (0.0235)	1.427*** (0.121)
Cons	4.667*** (0.0924)	0.118*** (0.0170)	5.535*** (0.0484)	4.873*** (0.0668)	0.0555*** (0.0161)	5.968*** (0.0491)
Year	YES	YES	YES	YES	YES	YES
N	5848	5848	5848	5848	5848	5848
R-squared	0.248	0.153	0.153	0.331	0.241	0.126

Note: *** p < 0.01, ** p < 0.05, * p < 0.10; Standard errors is in parentheses; N represents sample size.

Table 12 presents the heterogeneous mediation effects of zombie firms across regions. The findings reveal that the indirect negative impact of government subsidies on aggregate TFP through zombie firm formation differs between the Eastern and Central–Western regions. Specifically, the effect of government subsidies on zombie firm formation is slightly stronger in Central and Western China, while the negative impact of zombie firms on aggregate TFP is marginally larger in the Eastern region. This may reflect the generally lower productivity levels of firms in the Central and Western regions, which reduce the productivity gap between zombie and non-zombie firms. In contrast, the higher overall efficiency and stronger market competition in the East amplify the relative productivity disadvantage of zombie firms, leading to a slightly stronger negative effect on aggregate TFP.

**Table 12:** Regional Heterogeneity of the Mediating Effects of Zombie Firms in the Relationship Between Government Subsidies and Aggregate TFP.

Variables		East				Central-West		
		TFP	ZoShare	TFP		TFP	ZoShare	TFP
SubInt	−5.381*** (1.643)		3.027*** (0.283)		−4.777*** (1.660)	−10.64*** (1.330)	3.204*** (0.316)	−13.70*** (1.494)
ZoShare					−0.196** (0.0786)			−0.186*** (0.0593)
RDPers	2.763*** (0.212)		0.0370 (0.0423)		2.753*** (0.212)	0.560*** (0.188)	−0.0459 (0.0446)	2.667*** (0.189)
RDInv	−6.512*** (0.745)		−0.0774 (0.134)		−6.522*** (0.745)	−7.773*** (0.559)	0.0850 (0.133)	−5.945*** (0.607)
ALRatio	2.527*** (0.0900)		0.150*** (0.0155)		2.558*** (0.0908)	1.671*** (0.0679)	0.163*** (0.0161)	1.554*** (0.0764)
ROA	1.562*** (0.123)		−0.660*** (0.0215)		1.435*** (0.133)	1.651*** (0.0880)	−0.674*** (0.0209)	1.307*** (0.106)
Cons	5.472*** (0.0531)		0.0641*** (0.0153)		5.475*** (0.0530)	4.764*** (0.0714)	0.0858*** (0.0170)	5.890*** (0.0457)
Year	YES		YES		YES	YES	YES	YES
N	5436		5436		5436	6260	6260	6260
R-squared	0.177		0.195		0.178	0.302	0.199	0.124

Note: *** p < 0.01, ** p < 0.05, * p < 0.10; Standard errors is in parentheses; N represents sample size.

### Robustness test

To assess the robustness of our mediation results, we conducted two additional tests. Table 13 summarizes these robustness checks.First, we re-estimated the model using city–industry–year aggregated data to examine whether the results depend on the level of data aggregation.Second, we used lagged subsidy intensity to mitigate concerns regarding potential endogeneity and reverse causality.Across both robustness checks, the sign and significance of the mediation pathway remained consistent with the baseline model, confirming the stability of our findings.

**Table 13:** Robustness Checks for the Mediation Model.

Variables	Aggregated Mediation Regression (City–Industry–Year)			Mediation Regression with Lagged Subsidy Intensity		
	TFP	ZoShare	TFP	TFP	ZoShare	TFP
SubInt	−2.271*** (0.537)	1.790*** (0.118)	−2.014*** (0.539)			
L1_SubInt				−9.126*** (1.197)	1.687*** (0.473)	−8.853*** (1.206)
ZoShare			−0.144*** (0.0315)			−0.162*** (0.0557)
RDPers	−0.625*** (0.0946)	0.00139 (0.0208)	−0.625*** (0.0945)	2.725*** (0.175)	−0.0980*** (0.0312)	2.710*** (0.175)
RDInv	−3.072*** (0.166)	−0.00341 (0.0365)	−3.072*** (0.166)	−7.038*** (0.537)	0.219* (0.119)	−7.002*** (0.536)
ALRatio	2.304*** (0.0391)	0.131*** (0.00860)	2.323*** (0.0393)	1.942*** (0.0670)	0.169*** (0.0132)	1.970*** (0.0668)
ROA	1.417*** (0.0444)	−0.507*** (0.00979)	1.345*** (0.0472)	1.632*** (0.0879)	−0.628*** (0.0320)	1.530*** (0.0976)
*Cons*	4.491*** (0.0450)	0.0884*** (0.00992)	4.504*** (0.0451)	5.824*** (0.0396)	0.0135* (0.00764)	5.826*** (0.0396)
Year	YES	YES	YES	YES	YES	YES
N	20,817	20,818	20,817	10,078	10,078	10,078
R-squared	0.24	0.156	0.241	0.15	0.175	0.151

Note: *** p < 0.01, ** p < 0.05, * p < 0.10; Standard errors is in parentheses; N represents sample size.

## Concluding remarks and policy implications

This study investigates the mediating role of zombie firm formation in the relationship between government subsidies and aggregate TFP among Chinese enterprises. Our empirical findings yield several important insights:

First, government subsidies significantly contribute to the persistence and proliferation of zombie firms by reducing subsidized firms’ market exit probability. This negative outcome is particularly pronounced among SOEs and firms located in the less economically developed Central and Western regions. Despite the implementation of supply-side structural reforms intended to reduce inefficiency, zombie firms continue to represent a persistent and growing challenge, undermining efficient resource allocation and productivity growth.

Second, subsidies exert a negative impact on aggregate TFP, and zombie firms constitute an important mediating channel through which this adverse effect materializes. The mediating mechanism is substantially stronger among SOEs, reflecting their higher dependence on subsidies and greater survival of low-productivity firms. Regional differences arise primarily through distinct pathways: subsidies more strongly induce zombie formation in Central and Western regions, whereas zombie firms impose a larger productivity drag in the more competitive Eastern region.

Based on these findings, several policy implications emerge. First, subsidy allocation mechanisms should shift from broad-based support toward more selective, competitive, and performance-oriented designs. Targeting subsidies toward innovation, productivity enhancement, and capability development—rather than sustaining low-efficiency firms—can help ensure that subsidies contribute to rather than hinder productivity growth.

Second, strengthening market-oriented reforms, particularly in SOE governance and in regions with weaker institutional environments, is critical for preventing the persistence of zombie firms. Hardening budget constraints and improving regulatory discipline can effectively curb the survival of low-productivity firms and enhance aggregate efficiency.

Overall, the results highlight the need for dynamic and well-targeted government interventions. A balanced approach that considers both short-term economic stabilization and long-term productivity improvement is essential for achieving sustainable, high-quality economic development.

## Supporting information

S1 Data The dataset used in this article for discussion and analysis. (XLSX)
